# Human OAS1 activation is highly dependent on both RNA sequence and context of activating RNA motifs

**DOI:** 10.1093/nar/gkaa513

**Published:** 2020-06-17

**Authors:** Samantha L Schwartz, Esther N Park, Virginia K Vachon, Shamika Danzy, Anice C Lowen, Graeme L Conn

**Affiliations:** Department of Biochemistry, Emory University School of Medicine, 1510 Clifton Road NE, Atlanta, GA 30322, USA; Graduate Program in Biochemistry, Cell and Developmental Biology, Graduate Division of Biological and Biomedical Sciences, Emory University, USA; Department of Biochemistry, Emory University School of Medicine, 1510 Clifton Road NE, Atlanta, GA 30322, USA; Department of Biochemistry, Emory University School of Medicine, 1510 Clifton Road NE, Atlanta, GA 30322, USA; Graduate Program in Microbiology and Molecular Genetics, Graduate Division of Biological and Biomedical Sciences, Emory University, USA; Department of Microbiology and Immunology, Emory University School of Medicine, 1510 Clifton Road NE, Atlanta, GA 30322, USA; Graduate Program in Microbiology and Molecular Genetics, Graduate Division of Biological and Biomedical Sciences, Emory University, USA; Department of Microbiology and Immunology, Emory University School of Medicine, 1510 Clifton Road NE, Atlanta, GA 30322, USA; Department of Biochemistry, Emory University School of Medicine, 1510 Clifton Road NE, Atlanta, GA 30322, USA; Graduate Program in Biochemistry, Cell and Developmental Biology, Graduate Division of Biological and Biomedical Sciences, Emory University, USA; Graduate Program in Microbiology and Molecular Genetics, Graduate Division of Biological and Biomedical Sciences, Emory University, USA

## Abstract

2′-5′-Oligoadenylate synthetases (OAS) are innate immune sensors of cytosolic double-stranded RNA (dsRNA) and play a critical role in limiting viral infection. dsRNA binding induces allosteric structural changes in OAS1 that reorganize its catalytic center to promote synthesis of 2′-5′-oligoadenylate and thus activation of endoribonuclease L. Specific RNA sequences and structural motifs can also enhance activation of OAS1 through currently undefined mechanisms. To better understand these drivers of OAS activation, we tested the impact of defined sequence changes within a short dsRNA that strongly activates OAS1. Both *in vitro* and in human A549 cells, appending a 3′-end single-stranded pyrimidine (3′-ssPy) can strongly enhance OAS1 activation or have no effect depending on its location, suggesting that other dsRNA features are necessary for correct presentation of the motif to OAS1. Consistent with this idea, we also find that the dsRNA binding position is dictated by an established consensus sequence (WWN_9_WG). Unexpectedly, however, not all sequences fitting this consensus activate OAS1 equivalently, with strong dependence on the identity of both partially conserved (W) and non-conserved (N_9_) residues. A picture thus emerges in which both specific RNA features and the context in which they are presented dictate the ability of short dsRNAs to activate OAS1.

## INTRODUCTION

The innate immune system detects diverse foreign molecules, or pathogen-associated molecular patterns (PAMPs), to provide a critical line of defense against infection (1,2). Double-stranded RNA (dsRNA) is often a hallmark of viral infection, present in viral genomes or produced as a consequence of viral gene expression or replication (3–5). dsRNA is thus a potent PAMP detected by several distinct human cellular pattern recognition receptors, including the 2′-5′-oligoadenylate synthetase (OAS) family of cytosolic dsRNA sensors which restrict replication of multiple viruses (6,7).

In humans, the *OAS* family contains four genes that encode three catalytically active enzymes (OAS1, OAS2 and OAS3) and one catalytically inactive protein (OASL) (8). Activation of each catalytically active OAS enzyme by dsRNA leads to the non-processive synthesis of 2′-5′-linked oligoadenylate (2–5A) molecules which promote dimerization and activation of latent endoribonuclease L (RNase L) (9–12). Activated RNase L degrades single-stranded viral and cellular RNA, including specific mRNAs, tRNA and rRNA (13–18), thereby halting viral replication and limiting the spread of infection (19,20). The central importance of the OAS/RNase L pathway in control of infection is also reflected by the diverse array of viral mechanisms that inhibit this pathway or subvert the consequences of OAS activation (21–33).

OAS1 is capable of sensing short dsRNAs (>18 base pairs (bp)) and its antiviral role is supported by the identification of OAS1 single nucleotide polymorphisms (SNPs) important for determining susceptibility to West Nile virus infections (34–36). There is also accumulating evidence for essential roles played by OAS1 in normal cell function. For example, OAS1 SNPs have also been implicated in altered cellular function leading to diseases including diabetes (37), multiple sclerosis (38,39), prostate cancers (40), and Sjögren's syndrome (41,42), as well as susceptibility to tuberculosis infection (43). OAS1 has also been recently implicated in cancer cell survival after treatment with DNA damaging agents (44) and in mediating the cytotoxicity of 5-azacytidine (AZA), a DNA methyltransferase inhibitor widely used in cancer treatment (45). Taken together, these studies suggest important roles for OAS1 in both innate immunity and other cellular processes that we have yet to fully elucidate.

**Figure 1.**
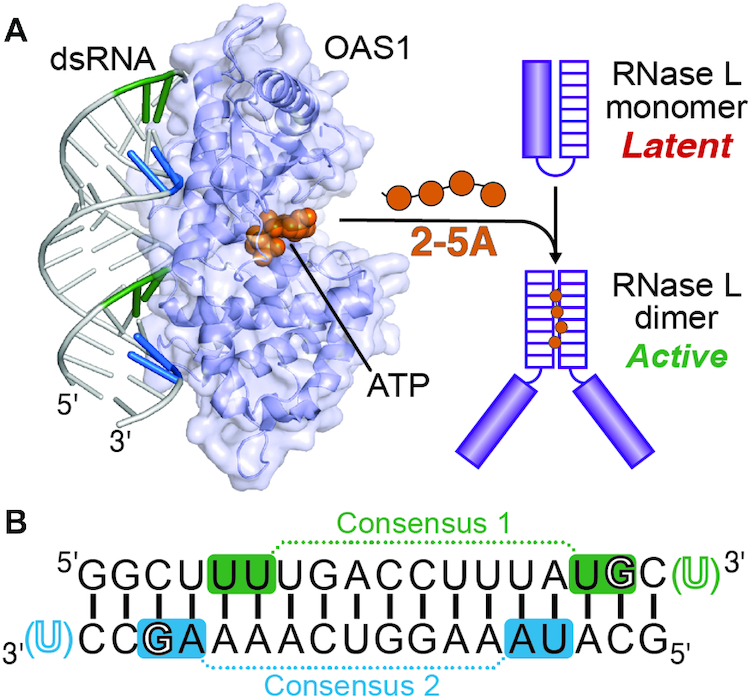
The OAS1/RNase L pathway and 18 bp dsRNA design. (**A**) Upon binding dsRNA (*left*), OAS1 (shown as a cartoon with semi-transparent surface; PDB ID 4IG8) produces 2′-5′-oligoadenylate molecules (2–5A; *orange*) which promote dimerization and activation of RNase L (*right*). (**B**) Sequence of the 18 bp dsRNA used in structural studies of the human OAS1-dsRNA complex (47) and as a starting point for the studies described here. This dsRNA contains two copies of a known OAS1 activation consensus sequence (WWN_9_WG) highlighted in both panels for Consensus 1 (*green*) and Consensus 2 (*blue*) on the top and bottom strands, respectively. The conserved consensus G nucleotide, which makes a base-specific contact with OAS1, and placements of the 3′-ssPy motif (*green* or *blue* U) used here in specific dsRNA constructs are also indicated with outline font.

OAS1 lacks a canonical RNA-binding motif and instead interacts with dsRNA through a relatively flat surface of positive residues on the opposite side of the protein from its ATP binding sites and catalytic center (46–48). dsRNA binding allosterically induces conformational changes in the OAS1 active site, driving polymerization of ATP into 2–5A (Figure 1A). Despite available OAS1 structural data, there is still relatively little known about how specific features of dsRNA contribute to the potency of OAS1 activation. Defining the contributions made by specific sequences and other RNA molecular signatures will be essential to fully elucidate how OAS1 is regulated by viral or cellular dsRNA.

OAS1 activation is strongly enhanced by at least two distinct activating consensus sequences (49,50): APyAPy(N)*n*CC, UU(N)*n*ACCC (where Py is C or U, and N is any nucleotide) and WWN_9_WG (where W is A or U). Our subsequent discovery of the 3′-end single-stranded pyrimidine (3′-ssPy) motif revealed that OAS1 activation can be further enhanced by single-stranded pyrimidine-rich (C or U) sequences appended to the 3′-end of a short (18 bp) activating dsRNA, as well as other more complex viral and cellular non-coding RNAs (51,52). The 3′-ssPy motif is naturally found on non-coding RNAs as a consequence of RNA polymerase III transcription termination (53), and we have previously speculated that such 3′-ends might also occur as a result of RNase L cleavage at preferred single-stranded UA or UU sequences within larger RNA molecules, further potentiating signaling via the OAS/RNase L pathway (51). In contrast, another recent study found that the 3′-ssPy motif had no effect on OAS1 activation (54). These conflicting observations suggest that the dsRNA context in which the 3′-ssPy motif is presented to OAS1 can affect its ability to enhance 2–5A synthesis and that, more generally, our understanding of how different activating motifs can combine for optimal OAS1 activation is incomplete.

The 18 bp dsRNA used in the human OAS1-dsRNA co-crystal structure (47) contains two overlapping, antiparallel copies of the WWN_9_WG consensus sequence (Figure 1B). The complexities of elucidating the contributions of activating motifs even within short dsRNAs are highlighted by the fact OAS1 bound this dsRNA in a single orientation despite the presence of two potential binding sites containing a preferred activation sequence. Here, the requirements for OAS1 activation by short dsRNAs are explored further using variations on this model 18 bp dsRNA. These studies reveal that binding orientation-specific activation also exists in solution and that OAS1 activation by short dsRNAs is unexpectedly sensitive to both RNA sequence and the relative organization of activating motifs.

## MATERIALS AND METHODS

### OAS1 protein expression and purification

Human OAS1 was expressed in *Escherichia coli* BL21(DE3) as an N-terminal 6xHis-SUMO fusion of amino acids 1–346 (corresponding to the common, core residues of all OAS1 splicing isoforms) from vector pE-SUMO (LifeSensors). Cells were grown in lysogeny broth (LB) at 37°C to mid-log phase (optical density at 600 nm of ∼0.5), and protein expression was induced with 0.5 mM isopropyl β-d-1-thiogalactopyranoside (IPTG) with continued growth overnight at 20°C. Cells were lysed by sonication in 50 mM Tris–HCl buffer (pH 8.0) containing 300 mM NaCl, 20 mM imidazole, 10% (v/v) glycerol and 10 mM β-mercaptoethanol. SUMO-OAS^1–346^ fusion protein was purified from cleared lysate by Ni^2+^-affinity chromatography on an ÄKTApurifier 10 FPLC system (GE Healthcare) and dialyzed overnight against SUMO cleavage buffer, containing 50 mM Tris–HCl (pH 8.0), 150 mM NaCl, 10% (v/v) glycerol and 2 mM DTT. The partially purified fusion protein was then stored at –80°C. Prior to each experiment, the N-terminal 6xHis-SUMO tag was cleaved by incubating SUMO-OAS^1–346^ fusion protein with SUMO protease (Ulp1) for 90 min at 30°C and an additional hour at 4°C, followed by dialysis against the appropriate assay buffer. This process produces OAS^1–346^ with a native N-terminus after SUMO tag removal.

### Generating 18 bp dsRNA duplexes

Each RNA strand was chemically synthesized (Integrated DNA Technologies) and used without further purification. Each 18 bp dsRNA duplex was generated by mixing individual strands at equimolar concentrations and annealing by heating to 65°C for 10 min followed by slow cooling to room temperature. Native PAGE (20% acrylamide in 0.5× Tris–borate–EDTA) was used to verify the homogeneity of both single-strand RNAs and dsRNA duplexes prior to use. Each lane contained RNA (100 ng total) resolved on gels run at 120 V for 3 h at 4°C, visualized by staining with SYBR Gold (1:10 000, Invitrogen), and imaged on a Typhoon Trio Imager (GE Healthcare).

### Chromogenic assay of OAS1 activity

OAS^1–346^ was dialyzed overnight against OAS1 activity assay buffer: 50 mM Tris–HCl (pH 7.4) containing 100 mM NaCl, 1 mM EDTA and 1 mM DTT. Pyrophosphate (PP_i_), the reaction by-product of 2–5A synthesis by OAS1, was monitored using a chromogenic assay adapted from previously established methods for measurement of OAS1 activity (51,52,55). OAS^1–346^ (100 nM) was incubated with 10 μg/ml poly(rI:rC) or 300 nM dsRNA in reactions containing final solution conditions of 25 mM Tris–HCl (pH 7.4), 10 mM NaCl, 7 mM MgCl_2_, 1 mM DTT and 2 mM ATP at 37°C in a 150 μl total reaction volume. Aliquots (10 μl) were removed over a 0–120 min time course and the reaction immediately quenched by adding directly to the wells of a 96-well plate pre-dispensed with 250 mM EDTA (pH 8.0, 2.5 μl). At completion of the time course, 2.5% (w/v) ammonium molybdate in 2.5 M H_2_SO_4_ (10 μl) and 0.5 M β-mercaptoethanol (10 μl) were added to each well and the final volume brought to 100 μl with water. Absorbance at 580 nm was measured using a Synergy Neo2 plate reader (BioTek). Readings were subtracted from background using an ATP-only control reaction (lacking both OAS^1–346^ and dsRNA) and then converted to pyrophosphate produced (nmols) using a pyrophosphate standard curve. Experiments were performed as four independent assays using two different preparations of OAS^1–346^, each comprising three technical replicates which were averaged prior to data analysis. Final values were plotted with their associated standard error of the mean (SEM) in GraphPad Prism 8.

Kinetic analyses were performed similarly but using a range of dsRNA concentrations (0.1–5 μM) and measuring PP_i_ production only for the initial 12 min of the reaction with two technical replicates for each experiment. Linear regression analysis was used to obtain initial rates of reaction (nmol PP_i_ produced/min) for each dsRNA concentration. These values were plotted and a non-linear regression analysis performed to obtain *V*_max_ and *K*_app_ values using the Michaelis–Menten model equation: *Y* = (*V*_max_*X*)/(*K*_app_ + *X*) in GraphPad Prism 8.

### OAS1/RNase L activation in A549 cells

Human wild-type and RNase L knock-out A549 cells, constructed using CRISPR-Cas9 gene editing technology as reported previously (6), were cultured in RPMI1640 cell culture medium supplemented with 10% fetal bovine serum and 100 μg/ml Normocin™. Both cell lines were monitored regularly and tested negative for mycoplasma. Cells were seeded into 12-well plates at 3 × 10^5^ cells/well and were treated with 5000 U/ml Interferon-α (Sigma) prior to dsRNA transfection. Following overnight interferon treatment (16 h), cells were transfected with dsRNAs (50 nM) or poly(rI:rC) (0.1 μg/ml) using siLentFect Lipid Reagent (BioRad) and incubated at 37°C for 6 h. Cells were lysed and total RNA was isolated using a RNeasy Plus Mini Kit (Qiagen) following manufacturer instructions. Total RNA was resolved using an Agilent 2100 Bioanalyzer system. At least two independent sets of experiments were performed with essentially identical results for both the wild-type (*n* = 3) and RNase L knock-out (*n* = 2) cells, respectively. Bands resulting from 28S rRNA cleavage in wild-type A549 cells were quantified using ImageJ and relative cleavage was calculated for each set of averaged technical replicates by normalizing to activity induced by the 18 bp dsRNA with no 3′-ssPy.

### OAS1-dsRNA 4-thiouridine (4-thioU) crosslinking

RNA strands containing a 3′-ssPy motif modified with a 4-thiouridine were chemically synthesized (Dharmacon), purified by high performance liquid chromatography (HPLC), and 2′-deprotected before use. RNA strands containing the 4-thiouridine modification (3′-end) were 5′-end labeled using [γ-^32^P]-ATP and T4 polynucleotide kinase (PNK), excess [γ-^32^P]-ATP was removed using an Illustra MicroSpin G-25 column (GE Healthcare), and radiolabel incorporation was quantified by scintillation counting. The radiolabeled 18 bp dsRNA duplexes were generated by mixing the radiolabeled strand (containing 4-thiouridine) with the same unlabeled strand and a slight total excess of unlabeled complement. The mixed strands were annealed by heating to 65°C for 10 min followed by slow cooling to room temperature. OAS^1–346^ was dialyzed overnight against OAS1 activity assay buffer but lacking DTT, i.e. 50 mM Tris–HCl (pH 7.4) containing 100 mM NaCl and 1 mM EDTA.

OAS^1–346^ (5 μM) was incubated with radiolabeled, 4-thiouridine containing 18 bp dsRNA duplex (500 nM) for 30 min on ice with UV exposure at 365 nm and using a 96-well plate format at a distance of 3 cm. Reactions were stopped by addition of SDS loading buffer and heating to 95°C for 5 min. Reaction products were resolved on a 10% SDS-PAGE run at 200 V for 45 min to allow for sufficient separation of OAS1–dsRNA complex and free RNA. Gels were soaked in destain solution (50:40:10% ethanol:water:acetic acid; 15 min), fixed (20% ethanol, 2% glycerol; 15 min), dried, and imaged using a Typhoon FLA 7000 PhosphorImager and ImageQuant software (GE Healthcare).

## RESULTS

### The 3′-ssPy motif enhances OAS1 activation only when appended to the *top* strand of the 18 bp dsRNA

Our previous analysis of OAS1 activation by the model 18 bp dsRNA revealed that a single 3′-end unpaired U nucleotide (3′-ssPy motif) appended to one strand of the dsRNA was sufficient to potentiate OAS1 activation *in vitro* (51). In this original study, the 3′-ssPy motif was placed on the ‘top’ RNA strand (as depicted in Figure 1B), corresponding to the dsRNA end closest to the base-specific interaction made by OAS1 (via the Ser56 backbone carbonyl group) to the activation consensus guanosine nucleotide (WWN_9_WG) in the OAS1–dsRNA complex crystal structure. However, the question remained as to whether addition of a 3′-ssPy motif to the complementary (‘bottom’) strand of this dsRNA could similarly enhance OAS1 activity. Enhanced activation by a 3′-ssPy on the bottom strand would indicate a lack of dsRNA binding preference or absence of orientation-specific activation in solution. In contrast, a different impact of a 3′-ssPy on the bottom strand would reveal a binding preference in solution similar to that observed in the human OAS1–dsRNA crystal structure. Thus, addition of a 3′-ssPy to either strand of this model dsRNA can serve as a tool to assess OAS1 sensitivity to dsRNA binding orientation and OAS1 activation in solution.

**Figure 2.**
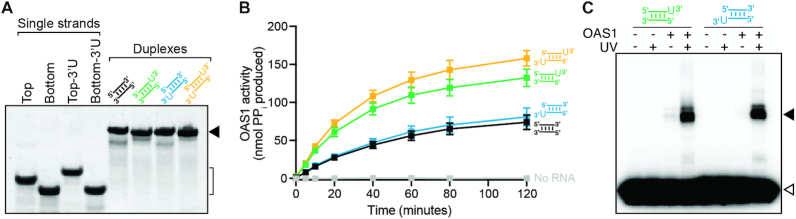
The 3′-ssPy motif impacts OAS1 activity *only* when appended to the top strand. (**A**) Native PAGE analysis showing purity of the individual chemically synthesized ssRNAs and stable formation of each dsRNA, indicated by the bracket and solid arrowhead on the right of the image, respectively. (**B**) Reaction progress curves from an *in vitro* chromogenic assay of OAS1 activity using a single dsRNA concentration (300 nM) for no 3′-ssPy (*black*), or with the motif appended to the top (*green*), bottom (*blue*) or both strands (*orange*). OAS1 activation is substantially enhanced only when the 3′-ssPy motif is placed on the top strand. (**C**) OAS1–dsRNA crosslinking induced by irradiation with UV light (365 nm) resolved by SDS-PAGE to separate crosslinked complex (*solid arrow*) from free RNA (*open arrow*). ssRNAs containing 4-thioU modifications were 5′-end labeled with ^32^P for visualization prior to annealing. Both dsRNAs crosslink to OAS1 via the 4-thioU regardless of 3′-ssPy motif location.

To address this open question, we generated and compared four 18 bp dsRNAs derived from the model dsRNA duplex used in the OAS1–dsRNA complex crystal structure (Figure 1): no 3′-ssPy motif, top strand only, bottom strand only, and on both strands. For these analyses, one 3′-end single-stranded U nucleotide was used to represent the 3′-ssPy motif as our previous study showed a single additional pyrimidine was sufficient and addition of U or C-rich sequences were functionally equivalent in their ability to enhance OAS1 activation (51). Each dsRNA was generated by annealing the corresponding single-strand RNAs and stable duplex formation confirmed by native polyacrylamide gel electrophoresis (PAGE; Figure 2A). Each dsRNA was then tested in an established *in vitro* OAS1 activation assay, which measures the amount of inorganic pyrophosphate (PP_i_) produced as a consequence of dsRNA-induced 2–5A synthesis. As previously observed (51), the level of OAS1 activity is significantly enhanced by addition of a top strand 3′-ssPy motif compared to the unaltered dsRNA (Table 1 and Figure 2B, compare green and black curves, respectively). In contrast, addition of a 3′-ssPy to the bottom strand had no effect on OAS1 activation (Table 1 and Figure 2B, compare blue and black curves), while its addition in the context of a top strand 3′-ssPy motif only modestly further increased activation (Table 1 and Figure 2B, compare orange and green curves). Thus, the 3′-ssPy motif only significantly impacts OAS1 activation by this short dsRNA when appended to the top strand.

**Table 1.** Summary of OAS1 activation by short dsRNAs *in vitro* and A549 cells

		Kinetic analysis^b^		
3′-ssPy location	Initial rate (nmol PP_i_/ min)^a^	*K*_app_ (μM)	*V*_max_ (nmol PP_i_/ min)	Relative 28S rRNA cleavage^c^
None	1.4 ± 0.1	10.3 ± 1.0	36.0 ± 2.5	1.0
Top	3.5 ± 0.3	4.0 ± 0.3	36.5 ± 1.4	1.7 ± 0.5
Bottom	1.6 ± 0.2	6.5 ± 0.6	22.8 ± 1.5	0.6 ± 0.2
Both ends	4.0 ± 0.2	ND	ND	2.7 ± 0.2
	Initial rate (nmol PP_i_/ min)^a^			
	Base Pair Move	Scramble (Consensus)		Consensus Strand Swap
None	2.0 ± 0.1	0.74 ± 0.05		0.68 ± 0.06
Top	2.4 ± 0.2	1.3 ± 0.1		1.0 ± 0.1
Bottom	2.7 ± 0.3	0.80 ± 0.04		0.64 ± 0.04
Both ends	2.8 ± 0.3	1.2 ± 0.1		0.95 ± 0.04

^a^Initial rates determined by linear regression analysis on the 0–10 min time points for replicate time course experiments performed at a single dsRNA concentration (300 nM dsRNA), as shown in Figures 2, 5, and 6.

^b^Kinetic parameters (*K*_app_ and *V*_max_) were determined from fits to data shown in Figure 4. ND, Not determined.

^c^Values from quantification of three independent experiments in wild-type A549 cells, exemplified in Figure 3.

To assess whether the 3′-ssPy location can affect dsRNA–OAS1 interaction, we used OAS1–dsRNA crosslinking by exploiting the presence of the 3′-ssPy motif to site-specifically incorporate the photoreactive crosslinker 4-thiouridine (4-thioU). The modified single uridine (3′-ssPy motif) was added individually to each end of the dsRNA to generate top or bottom strand 4-thioU 18 bp dsRNAs which were crosslinked to OAS1 (present in 10 × excess) by exposure to 365 nm UV light. In each dsRNA, the RNA strand containing the 4-thioU modification was also 5′-end ^32^P-labeled for visualization by autoradiography. Crosslinking and control reactions were resolved by SDS-PAGE to separate crosslinked OAS1–dsRNA complexes from free dsRNA (Figure 2C), revealing both 3′-ssPy-containing dsRNAs to be specifically, and to similar extents, crosslinked to OAS1 upon UV light treatment (Figure 2C, lanes 4 and 8). Therefore, the observed differences in OAS1 *in vitro* activity for these two dsRNAs (Table 1 and Figure 2B; green and blue) do not appear to be due to significant differences in the nature or extent of OAS1–dsRNA complex formation (Figure 2C).

### Short dsRNAs activate the OAS1/RNase L pathway in human A549 cells mirroring their capacity to activate OAS1 *in vitro*

We next tested the ability of the same four 18 bp dsRNAs to activate the OAS1/RNase L pathway in human lung carcinoma A549 cells as assessed by RNase L-mediated rRNA cleavage. A549 cells were selected for these experiments because they express OAS1 well, are amenable to transfection with small RNAs, and are the same background used to generate an RNase L CRISPR–Cas9 knock-out cell line (6). Because the model dsRNAs are only 18 bp in length, they are too short to activate either OAS2 or OAS3 (54,56,57) and must therefore act exclusively via OAS1 to promote RNase L activation. For these experiments, A549 cells were transfected with one of the four dsRNAs, containing (or lacking) 3′-ssPy motif(s), in parallel with poly(rI:rC) dsRNA transfection and mock transfected cells, as positive and negative controls, respectively.

**Figure 3.**
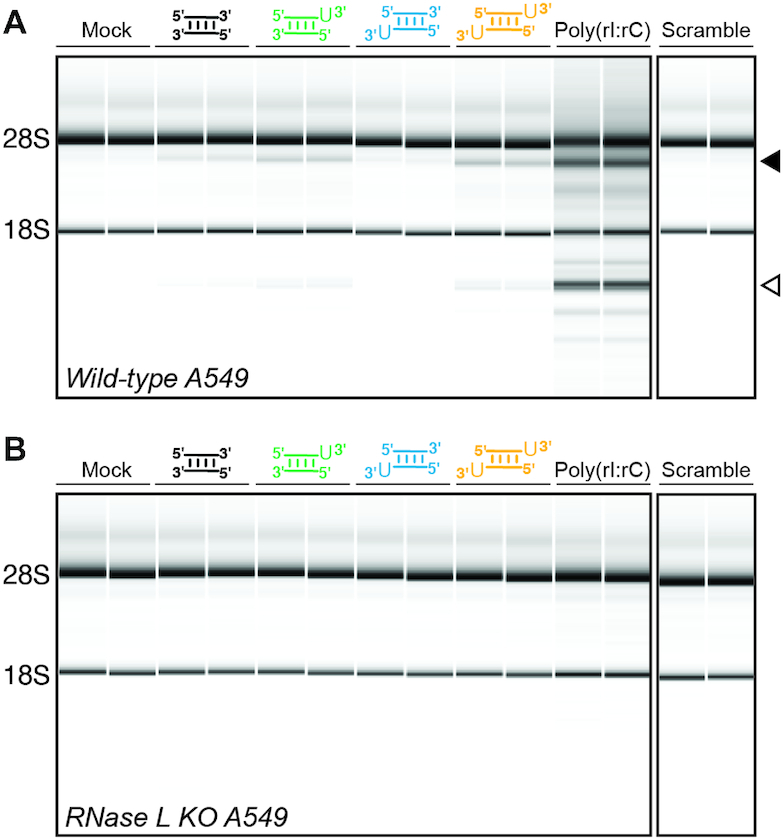
OAS/RNase L pathway activation in A549 cells by the short dsRNAs correlates with their ability to activate OAS1 *in vitro*. (**A**) Bioanalyzer analysis of rRNA integrity in A549 cells following transfection with the indicated dsRNAs (18 bp dsRNAs with 3′-ssPy motifs appended as indicated and an 18 bp Scramble dsRNA). Mock transfection and transfection with poly(rI:rC) dsRNA serve as negative and positive controls, respectively. OAS1/RNase L pathway activation is indicated by 28S and 18S rRNA degradation (*arrows*). Quantification of the 28S rRNA cleavage product (*solid arrow)* is given in Table 1. (**B**) As for *panel A* but using A549 RNase L KO cells (6). In both panels, a representative analysis with technical duplicates for each RNA is shown for one of at least two independent experiments which showed essentially identical results.

Transfection with each dsRNA duplex or poly(rI:rC) dsRNA resulted in rRNA degradation (Figure 3A and Table 1) compared to the mock-transfected control and consistent with activation of the OAS1/RNase L pathway. Further, dsRNAs containing the 3′-ssPy on the top strand or on both strands (Figure 3A, green and orange, respectively) resulted in more cleavage overall compared to the dsRNAs containing 3′-ssPy on the bottom strand or lacking a 3′-ssPy (Figure 3A, blue and black, respectively). Thus, these cell-based assays of OAS1 activation correlate well with the respective capacity of each dsRNA to activate OAS1 in the *in vitro* activity assay. Additionally, a dsRNA with identical nucleotide content to the 18 bp dsRNA but with different sequence (‘Scramble’ dsRNA) completely failed to activate RNase L, also consistent with its *in vitro* activity (see below). The higher overall rRNA cleavage observed for poly(rI:rC) dsRNA is likely due to its higher potency and additional capacity to activate OAS2 and OAS3 (58). Furthermore, dsRNA-mediated rRNA cleavage activity is completely absent in A549 cells lacking RNase L (RNase L KO A549; Figure 3B) confirming that the observed rRNA cleavage by the dsRNAs results exclusively from activation of the OAS/RNase L pathway via OAS1.

The results of both the *in vitro* and cell-based assays are consistent with either an OAS1 binding orientation preference for the 18 bp dsRNA (like that observed in the crystal structure), or a difference in ability of the dsRNA to activate OAS1 when bound in each of its two possible orientations. Either scenario is surprising, given that each strand of the dsRNA contains a sequence that conforms to the WWN_9_WG activation consensus sequence. However, the two potential binding orientations and the consensus sequence they each present to OAS1 are apparently non-equivalent in either their ability to bind OAS1, activate OAS1, or both.

### The 3′-ssPy placement can alter apparent dsRNA affinity and maximal OAS1 activation

**Figure 4.**
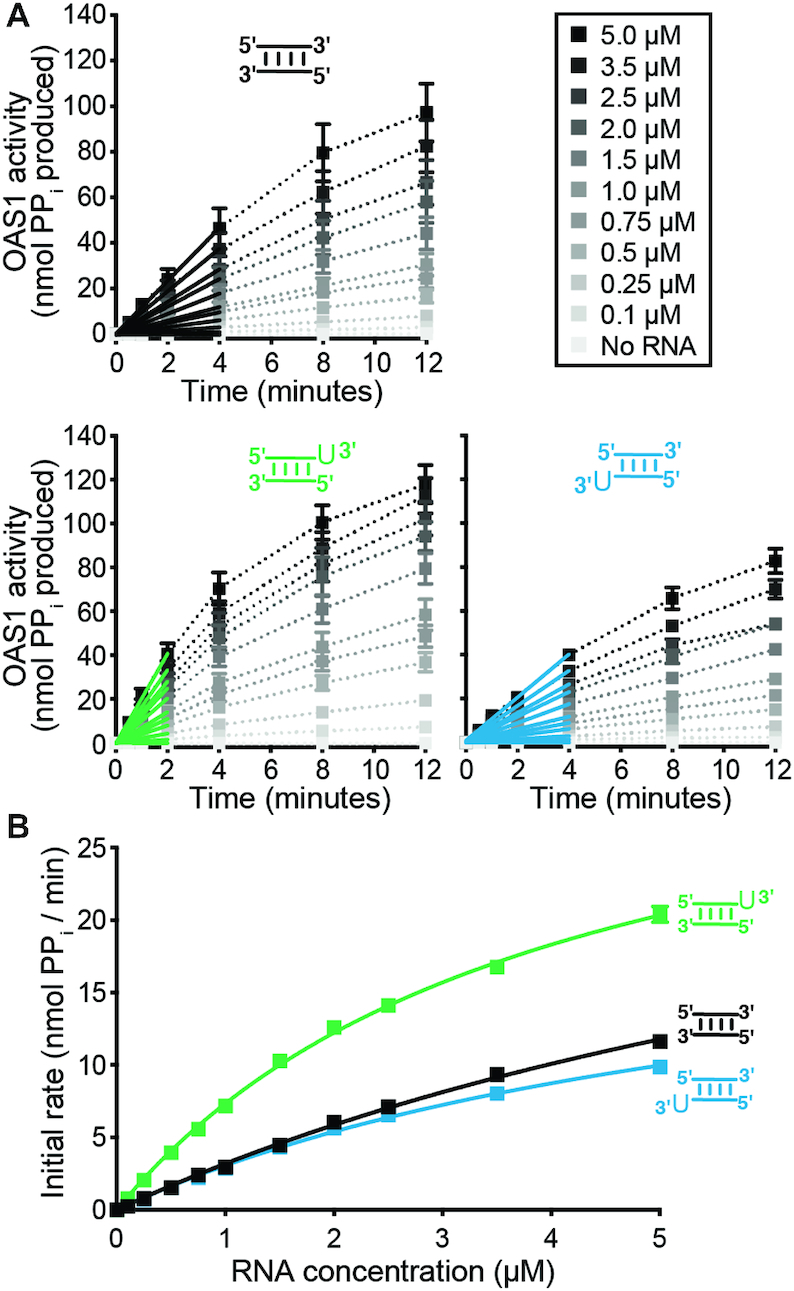
The 3′-ssPy motif differentially alters the kinetics of OAS1 activation when appended to the top or bottom strand. (**A**) Progress curves for dsRNA containing no (*black*), top (*green*), or bottom (*blue*) 3′-ssPy motifs over a range of dsRNA concentrations (0–5 μM) to determine initial rates of pyrophosphate (PP_i_) production. Data were fit using linear regression analysis for the first 2–4 min of the reaction (*color-coded solid lines*) in order to obtain the initial rate. (**B**) Kinetic analyses using calculated initial rates from *panel A* for OAS1 activation by each dsRNA variant. Data were fit using non-linear regression analysis to obtain the *V*_max_ and *K*_app_ values shown in Table 1. All error bars indicate standard error of the mean (SEM) and may not be visible if their width is smaller than the data point used.

To define the basis of the differing capacity of the 18 bp dsRNAs to activate OAS1, we used the *in vitro* OAS1 activity assay to assess enzyme activation over a wide range of dsRNA concentrations. Initial rates of reaction at each dsRNA concentration were determined and used to derive the maximum reaction velocity (*V*_max_) and apparent RNA dissociation constant (*K*_app_), as a measure of OAS1 catalytic activity and proxy for dsRNA binding affinity, respectively (Figure 4A,B and Table 1). Both dsRNAs containing the motif exhibited a decreased K_app_, corresponding to higher apparent dsRNA affinities, of 2.5-fold (top strand 3′-ssPy; Figure 4B, green) and 1.5-fold (bottom strand 3′-ssPy; Figure 4B, blue) compared to the dsRNA completely lacking a 3′-ssPy (Figure 4B, black). In contrast, while there was no measurable change in *V*_max_ with a top strand 3′-ssPy, we observed a modest decrease (1.5-fold) for a bottom strand placement of the motif. Therefore, the differences in OAS1 activity for these dsRNA duplexes appear to be due, at least in part, to changes in both apparent RNA affinity and maximum rate of 2–5A produced by OAS1 in the presence of each dsRNA. Specifically, a top strand 3′-ssPy reduces the dsRNA concentration required to reach maximal OAS1 activation but does not influence OAS1 *V*_max_. In contrast, a bottom strand 3′-ssPy increases apparent dsRNA affinity (albeit to a lesser extent) and reduces the maximal activation of the enzyme. Thus, the 3′-ssPy appears to have context-dependent impacts on the kinetics of OAS1 activation.

### Altering the distance between the 3′-ssPy motif and the consensus sequence impacts its ability to enhance OAS1 activation

**Figure 5.**
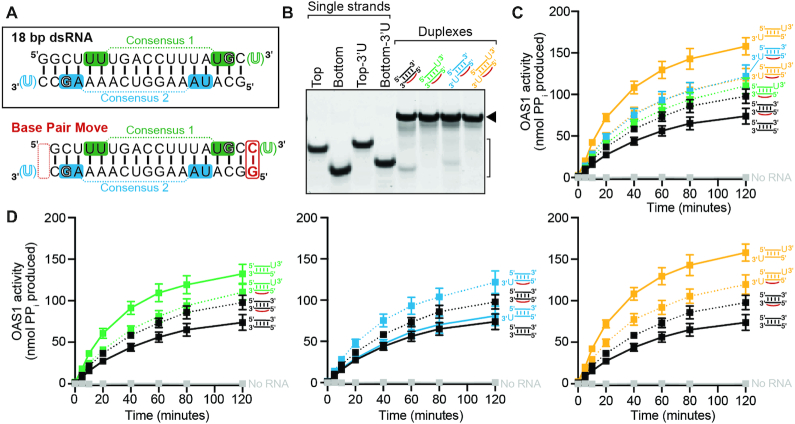
The relative context of the 3′-ssPy motif and consensus sequence is important for OAS1 activation. (**A**) Schematic of the base pair move dsRNA which is altered compared to the original 18 bp dsRNA by movement of a single G–C base pair from one end (*left, dotted red line*) to the other (*right, solid red line*). The original 18 bp dsRNA is shown (*black box*) for comparison. (**B**) Native gel analysis showing purity of ssRNAs (*bracket*) and stable formation of each dsRNA (*solid arrow*). (**C**) OAS1 enzyme progress curves comparing all base pair move dsRNAs (*dotted lines*); curves for the original 18 bp dsRNA with no 3′-ssPy (*solid black line*) and 3′-ssPy appended on both strands (*solid orange line*) are the same as in Figure 2B and are shown for comparison to indicate the reduced overall range of activation by the base pair move dsRNAs. (**D**) OAS1 enzyme progress curves are shown as pair-wise comparisons for each 3′-ssPy variant in both the 18 bp dsRNA (*solid lines*) and base pair move (*dotted lines*) contexts: no 3′-ssPy (*black*), top stand only (*green*), bottom strand only (*blue*), and on both strands (*orange*). The No RNA control (*gray*) is also shown in each panel.

We next asked whether the proximity of each consensus sequence to its corresponding 3′-end is the critical factor in determining the ability of a 3′-ssPy at each location on the 18 bp model dsRNA to enhance OAS1 activation. Specifically, the top strand WWN_9_WG consensus and adjacent 3′-ssPy are separated by one base pair, while the equivalent sequences on the bottom strand are separated by two base pairs. We therefore generated a new 18 bp dsRNA in which the additional G-C base pair separating the terminal G of the consensus sequence on the lower strand from the 3′-end of the RNA was ‘moved’ to the opposite end of the dsRNA (Figure 5A). Thus, this new dsRNA context (‘Base Pair Move’) has an increased distance between the top strand consensus sequence and its corresponding 3′-ssPy motif (by +1 bp) and an equivalent decrease in this distance for the bottom strand (by –1 bp), while maintaining the length of the dsRNA as 18 bp. We predicted that if the relative spacing of the consensus sequence and 3-ssPy is critical that we should observe an increase in activation resulting from a 3′-ssPy placed on the bottom strand, and a concomitant decrease in the effect conferred by the motif when appended on the top strand.

A set of four equivalent dsRNAs with the G-C base pair moved was generated (i.e. with no 3′-ssPy, top strand only, bottom strand only, and both strands) and each dsRNA was evaluated by native PAGE, as before (Figure 5B). The movement of this single base pair had two unanticipated effects on the extent of OAS1 activation, both modestly increasing activation in the absence of any appended 3′-ssPy while also decreasing activation by the most potent dsRNA, i.e. with the motif on both strands (Table 1 and Figure 5C, black and orange curves, respectively). Pairwise comparisons of each 3′-ssPy addition in the context of both the 18 bp dsRNA and base pair move dsRNA were conducted to determine whether the resulting activation level of OAS1 was altered in a manner corresponding with the spacing between each consensus and its associated 3′-end. As anticipated, addition of a 3′-ssPy to the bottom strand of the base pair move dsRNA resulted in increased OAS1 activation (Figure 5D, *center*). In contrast, addition of the motif to the top strand of the Base Pair Move dsRNA (now with increased spacing between the consensus and 3′-ssPy) no longer increased OAS1 activation (Figure 5D, *left*). Addition of a 3′-ssPy motif to both strands of the Base Pair Move dsRNA resulted in no further activation of OAS1 compared to addition to the bottom strand only, (Figure 5C and D, *right*), confirming the switch in 3′-ssPy sensitivity to the bottom strand in this altered dsRNA context.

These data thus support the idea that the nucleotide spacing between the consensus sequence and a 3′-ssPy appended to the end of the same strand is important for the latter motif's optimal placement and thus ability to potentiate OAS1 activation. These observations also suggest that OAS1 binds the 18 bp dsRNA in both orientations but that these binding modes do not result in equivalent OAS1 activation. Furthermore, the unexpected changes in activation with the single G–C base pair movement, which resulted in a narrower range of OAS1 activation, also suggest there are other elements of RNA sequence, in addition to the relative placement of the consensus sequence and 3′-ssPy, that contribute to the ability of a given dsRNA to activate OAS1.

### OAS1 activation consensus sequence nucleotide identities (WW/WG) and their placement within a dsRNA are important for OAS1 activation

**Figure 6.**
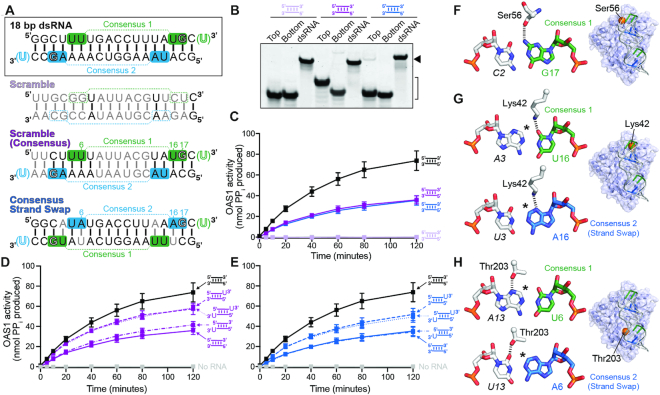
The OAS1 activation consensus sequence (WWN_9_WG) and its placement within dsRNA are also important for OAS1 activation. (**A**) Schematics of the additional 18 bp dsRNAs indicating the sequence changes to create the Scramble (nucleotides with altered identity are in *gray*), Scramble (Consensus), and Consensus Strand Swap. The original 18 bp dsRNA is shown (*black box*) for comparison. (**B**) Native gel analysis showing purity of ssRNAs (*bracket*) and stable formation of each dsRNA (*solid arrow*). (**C**) Progress curves for OAS1 activation by the Scramble (*mauve*), Scramble (Consensus) (*purple*), and Consensus Strand Swap (*blue*) dsRNAs compared to the original 18 bp dsRNA (*black*). (**D**) Progress curves for OAS1 activation for the Scramble (Consensus) with each possible 3′-ssPy variation (i.e. none, top, bottom, or both strands). (**E**) same as in *panel D*, except for the Consensus Strand Swap dsRNA. (**F–H**) Views of the base pair interactions of consensus sequence nucleotides (WW^6^/^16^WG^17^) directly contacted by OAS1 residues in the human OAS1-dsRNA structure (PDB 4IG8). The location of each OAS1 residue on the dsRNA binding surface is shown on a cartoon with semi-transparent surface rendering of the OAS1-dsRNA complex structure (indicated with an enlarged orange sphere at the residue's C_α_ atom). The view shown for each residue is related to that of Figure 1A by a 90° rotation around the y-axis. The asterisk in *panels G* and *H* denotes the approximate position of additional atoms of the exocyclic amino group that would be present if the A–U base pairing shown was replaced by G-C. Predicted interactions with Consensus 2 nucleotides in *panels G* and *H* were generated by mutation of the RNA sequence in PyMOL.

Given the unexpected sensitivity of the 18 bp dsRNA to the movement of a single base pair, we next asked whether the specific nucleotides within the consensus sequence (i.e. WW/WG and the intervening N_9_ spacer) also influence the extent of OAS1 activation. Three new dsRNA constructs were designed (Figure 6A): (i) a ‘Scramble’ dsRNA in which the order of most (15 of 18) nucleotides is altered while maintaining the nucleotide content of each strand; (ii) a ‘Scramble (Consensus)’ dsRNA in which the top and bottom strand WW/WG consensus nucleotides of the original 18 bp dsRNA are maintained within this new scrambled background and (iii) a ‘Consensus Strand Swap’ variant in which only the top and bottom strand WW/WG consensus nucleotides of the original 18 bp dsRNA are exchanged. Collectively, these new dsRNAs were designed to test the influence of overall dsRNA sequence, the nucleotides flanking the consensus sequences, and the specific nucleotide identities of the two different consensus sequences. In all three new dsRNA constructs, additional changes were made as required to maintain perfect Watson–Crick dsRNA base pairing (Figure 6A, B).

Remarkably, the Scramble dsRNA was completely unable to activate OAS1 *in vitro* (Figure 6C; mauve and gray curves, respectively) or in A549 cells (Figure 3), underscoring the unexpectedly strong sensitivity of OAS1 to specific dsRNA sequence. Returning the nucleotides (WW/WG) of the two OAS1 consensus sequences to their original locations within this new dsRNA background restored OAS1 activation, but not to the same extent as for the original dsRNA sequence (Figure 6C; compare purple and black curves, respectively). In this partially restored dsRNA context, we also tested the impact of addition of the 3′-ssPy motif on OAS1 activation (i.e. appended to top or bottom strand only, or to both strands; Figure 6D and Table 1). Consistent, with the earlier experiments in the original dsRNA construct, OAS1 activation is enhanced by the 3′-ssPy only when appended on the top strand. These experiments reveal that while the presence of the WW/WG dinucleotide pairs and their specific location are important for OAS1 activity, additional features also contribute to optimal OAS1 activation. Specifically, given the identical placement of the top strand consensus in each dsRNA, these results point to an unexpected influence of the ‘N_9_’ sequence identity for OAS1 activation, raising the question of how these nucleotides may exert an effect on OAS1 activity.

Finally, we similarly assessed the activity of the Consensus Strand Swap construct, which possesses the original N_9_ internal sequence but with the two consensus sequence WW/WG nucleotides on each strand switched (Figure 6A). This dsRNA also did not possess the same capacity to activate OAS1 as the original dsRNA (Figure 6C; compare blue and black curves, respectively), but resulted in an intermediate activation level, similar to that observed for the Scramble (Consensus) dsRNA. As before, addition of each potential combination of 3′-ssPy motifs on this dsRNA only showed enhanced OAS1 activation when the 3′-ssPy was appended on the top strand (Figure 6E and Table 1). These data indicate that the features which drive OAS1–dsRNA interaction and binding orientation-dependent OAS1 activation are conserved among these distinct dsRNA contexts. In particular, these results suggest that recognition of the conserved guanosine nucleotide (WW/WG), as previously observed in the OAS1–dsRNA complex structure, is a critical determinant of binding register (i.e. OAS1 location on the dsRNA helix) and thus optimal interaction and OAS1 activation. Additionally, examination of the three direct base interactions made by OAS1 to these base pairs in the human OAS1-dsRNA complex crystal structure (Figure 6F-H) allows rationalization of the differing abilities of UU/UG (Consensus 1) and UA/AG (Consensus 2) sequences to activate OAS1 (see Discussion).

## DISCUSSION

Nucleic acid sensing is an essential innate immune strategy for pathogen detection and initiation of downstream antiviral responses. Recent work has extended ideas of simple dsRNA length limitations for OAS protein activation (57), identifying sequence-specific motifs and activation sequences that can strongly enhance activation of the cytosolic dsRNA sensor OAS1 (49–52). However, the basis of their activity and whether they can act in concert (or competition) to control OAS1 activation is unknown. Fully defining these dsRNA features and their optimal contexts for OAS1 activation by viral or cellular RNAs is important because differing levels of pathway activation likely underpin regulation of distinct cellular processes or cell fates (e.g. apoptosis in response to viral infection).

Here, we used a model 18 bp dsRNA (47) to begin defining how specific RNA features affect OAS1 activation in a short dsRNA with more than one potential binding site. These studies revealed three key findings: (i) OAS1 is unexpectedly sensitive to RNA sequence as two dsRNAs of identical length and nucleotide content, but different sequence, could produce strong activation or no activation at all (18 bp dsRNA and Scramble dsRNA, respectively); (ii) interactions with the conserved nucleotides of the known activation consensus sequence (WWN_9_WG) (50) can dictate the specific register of binding on the dsRNA helix and (iii) only when appropriately positioned by these interactions can other activating motifs, such as 3′-ssPy, exert their effect on OAS1 activation. Additionally, we found that while the short dsRNA likely binds in both orientations in solution, unlike in the crystal structure (47), these two modes of OAS1 interaction are non-equivalent in their ability to promote 2–5A synthesis.

**Figure 7.**
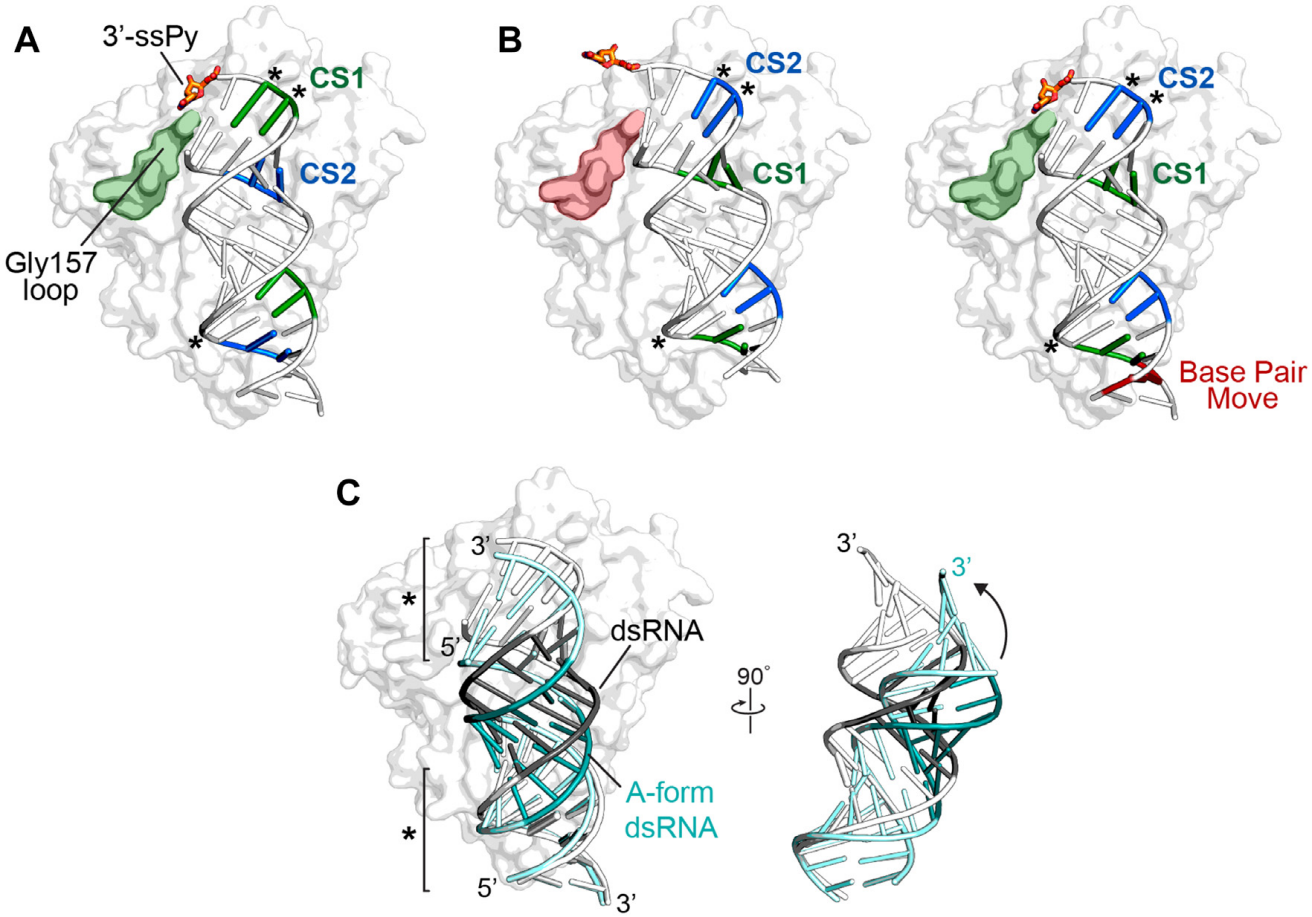
Models of OAS1 interactions with 18 bp dsRNA variants and the 3′-ssPy motif. (**A**) Structure of the OAS1-dsRNA complex (PDB 4IG8) with a top strand 3′-ssPy motif (*orange sticks*) modeled as previously reported (51). The consensus sequences on the top (CS1; *green*) and bottom (CS2; *blue*) strands of the dsRNA, and the OAS1 loop containing Gly157 (*green*) are shown on the dsRNA cartoon and OAS1 surface, respectively. (**B**) As in *panel A* but for the opposite dsRNA binding orientation, maintaining identical direct interactions between OAS1 and the dsRNA consensus nucleotides (marked *). The models shown are for the unaltered dsRNA in which the 3′-ssPy is no longer positioned to interact with the Gly157 loop (*left, red*) and the Base Pair Move dsRNA (*right*). (**C**) Comparison of the dsRNA in the OAS1 complex structure with a regular A-form dsRNA helix generated using the 3DNA webserver (60). The regions marked * are the main protein-RNA interaction surfaces.

OAS1 binds dsRNA via a relatively flat protein surface that spans approximately the length of an 18 bp dsRNA helix. This surface is enriched with basic residues that contact the sugar-phosphate backbone at each end of the dsRNA but make relatively few contacts to the center of the RNA helix (47). A number of base-specific interactions are also observed in the structure, including those made by Lys42, Ser56 and Thr203 with three conserved nucleotides of the activating WWN_9_WG consensus sequence (Figure 6F–H). These interactions appear to define the optimal register of binding on the dsRNA helix. Thus, when bound in the opposite orientation from that observed in the crystal structure, i.e. to engage the bottom strand consensus, the shift in register by one base pair needed to maintain interactions with the consensus sequence would likely weaken contacts with the lower half of the dsRNA helix (Figure 7), resulting in inefficient allosteric promotion of 2–5A synthesis.

Our previous modeling and mutagenesis studies suggested that the top strand 3′-ssPy motif interacts with an OAS1 loop containing residue Gly157 on the protein surface adjacent to the bound dsRNA (Figure 7A). Substitution of this residue resulted in loss of sensitivity to the 3′-ssPy but with otherwise the same activity as the wild-type enzyme in response to dsRNA binding (51). Following the finding here that 3′-ssPy motifs on each end of the dsRNA are sensed differently by OAS1, we modeled the interaction of the 18 bp dsRNA in its opposite orientation while maintaining the three direct OAS1-dsRNA base contacts to the bottom strand consensus sequence noted above (Figures 6F–H and 7B, *left*). In this orientation, the additional base pair between the bottom strand consensus sequence guanosine and its 3′-end shifts the modeled position of the 3′-ssPy such that it can no longer interact with the OAS1 Gly157 loop (Figure 7A, B) (51). However, when one base pair is removed, reducing the distance between the bottom strand consensus guanosine and the 3′-ssPy, the ability of the bottom 3′-ssPy motif to interact with the Gly157 would be restored (Figure 7B, *right*), consistent with the observed enhancement of OAS1 activation by the bottom strand 3′-ssPy in the context of the Base Pair Move dsRNA.

The 3′-ssPy thus appears to have context-dependent impacts on OAS1 activation: only when appropriately positioned by other dsRNA signatures, in this case by the precise binding register conferred by the activation consensus sequence, can the motif exert its effect on activation. This finding can readily explain the observation in another study using dsRNAs of various lengths (19–123 bp) that the 3′-ssPy motif had no effect on OAS1 activity (54): the dsRNAs used contained multiple WWN_9_WG consensus sequences, however, none were located equivalently to the top strand of the 18 bp dsRNA to optimally position the motif to interact with the Gly157 loop. The same study also found no effect of the 3′-ssPy motif on OAS2 activity, but whether similar constraints influence optimal positioning of the dsRNA in this protein (or OAS3), or OAS2 is simply insensitive to the motif will require further investigation. Conversely, there are examples of larger viral and cellular non-coding RNAs whose ability to activate OAS1 is enhanced by the 3′-ssPy motif in the absence of a ‘correctly’ positioned consensus sequence (51), suggesting that there are other unrelated sequences that also allow for optimal positioning of OAS1.

Our OAS1 kinetics analyses showed that placement of the 3′-ssPy on the bottom strand of the 18 bp dsRNA both increases apparent dsRNA affinity but also, unexpectedly, reduces the maximal activation of the enzyme compared to either no 3′-ssPy or top strand 3′-ssPy. We note that this effect is consistent with the bottom strand 3′-ssPy promoting increased interaction with an OAS1 binding site that results in weaker OAS1 activation. The two-fold difference in *V*_max_ observed for the 18 bp dsRNA with 3′-ssPy on the bottom strand could thus be explained by an ability to bind the dsRNA approximately half of the time in the ‘incorrect’ (or less productive) orientation. Further careful experiments will be required to fully test this concept of competing weaker or non-activating sites and the molecular basis of their action on OAS1. In the context of viral infection, for example, such insights would be important as a preferred OAS1 binding site in viral dsRNA with an intrinsically low ability to activate OAS1 could act as a ‘sponge’ to diminish activation by other sites within the same dsRNA (59).

Finally, the intermediate activation induced by both the Scramble (Consensus) and Consensus Strand Swap dsRNAs indicates that both the specific identity of the partially conserved (W = A/U) consensus nucleotides as well as the intervening (N_9_) sequence can strongly influence OAS1 activation. The role of the conserved WW/WG nucleotides can be partly rationalized based on interactions observed in the OAS1–dsRNA crystal structure. While the interaction with the consensus G nucleotide is conserved, different interactions are made at the other two positions directly contacted by OAS1 where the A–U base pair is reversed in both cases (Figure 6F–H). While Lys42 may be sufficiently flexible to make optimal interactions with A or U in the consensus sequence (though not a G–C pair with the additional amino group this would place in the dsRNA minor groove), we speculate that the closer placement of the U13 carbonyl group to Thr203 may be less readily accommodated, reducing OAS1 activation. The role of the intervening sequence is less easily rationalized but the dsRNA in the co-crystal structure is distorted from a perfect A-form helix, adopting a bent conformation to allow both ends of the dsRNA to contact OAS1 (Figure 7C). Consistent with the limited RNA-protein interactions in the center of the dsRNA helix, OAS1 is highly tolerant of GU-wobble pairs in the intervening region (N_9_) (47). We speculate that, rather than the nine non-conserved nucleotides being present simply to provide adequate spacing to place the WW and WG consensus nucleotides on the same face of the A-form helix (50), the specific sequence in this region also plays an indirect but important role in OAS1 activation by defining the dsRNA shape and flexibility upon protein binding.

The current work emphasizes the unexpectedly important role of RNA sequence and the context of activating motifs in OAS1 activation. While these facets of dsRNA-mediated OAS1 activation have been underappreciated to date, our findings are perhaps unsurprising given that OAS1–dsRNA interaction must be controlled to avoid aberrant activation by numerous cellular RNAs with sufficiently long dsRNA regions (47). These findings require us to begin redefining what constitutes a preferred sequence for promoting potent OAS1 activation and suggest there may be a continuum of activity with diverse sequences that fit a more general consensus. Further, the complexities revealed here in the context of a ‘simple’ model dsRNA are likely to become substantially more complicated in larger dsRNA molecules with multiple potential overlapping OAS1 binding sites. More precisely defining preferred OAS1 activation sequences would facilitate accurate location of these features in specific viral or cellular RNAs to provide deeper insights into both OAS1 regulation and its roles in the uninfected cell. Further work is thus needed to identify and characterize additional sequence-specific determinants, how these RNA features work in concert or in competition, as well the underlying molecular mechanism(s) by which they regulate OAS1 activation.

## References

[1] Schneider W.M., Chevillotte M.D., Rice C.M. Interferon-stimulated genes: a complex web of host defenses. Annu. Rev. Immunol. 2014; 32:513–545. 24555472 10.1146/annurev-immunol-032713-120231 PMC4313732

[2] Chow J., Franz K.M., Kagan J.C. PRRs are watching you: localization of innate sensing and signaling regulators. Virology. 2015; 479–480:104–109. 10.1016/j.virol.2015.02.051 PMC4424080 25800355

[3] Wu J., Chen Z.J. Innate immune sensing and signaling of cytosolic nucleic acids. Annu. Rev. Immunol. 2014; 32:461–488. 24655297 10.1146/annurev-immunol-032713-120156

[4] Schlee M., Hartmann G. Discriminating self from non-self in nucleic acid sensing. Nat. Rev. Immunol. 2016; 16:566–580. 27455396 10.1038/nri.2016.78 PMC7097691

[5] Hur S. Double-stranded RNA sensors and modulators in innate immunity. Annu. Rev. Immunol. 2019; 37:349–375. 30673536 10.1146/annurev-immunol-042718-041356 PMC7136661

[6] Li Y., Banerjee S., Wang Y., Goldstein S.A., Dong B., Gaughan C., Silverman R.H., Weiss S.R. Activation of RNase L is dependent on OAS3 expression during infection with diverse human viruses. Proc. Natl Acad. Sci. U.S.A. 2016; 113:2241–2246. 26858407 10.1073/pnas.1519657113 PMC4776461

[7] Schwartz S.L., Conn G.L. RNA regulation of the antiviral protein 2′-5′-oligoadenylate synthetase. WIREs RNA. 2019; 10:e1534. 30989826 10.1002/wrna.1534 PMC6585406

[8] Kristiansen H., Gad H.H., Eskildsen-Larsen S., Despres P., Hartmann R. The oligoadenylate synthetase family: an ancient protein family with multiple antiviral activities. J. Interferon Cytokine Res. 2011; 31:41–47. 21142819 10.1089/jir.2010.0107

[9] Dong B., Xu L., Zhou A., Hassel B.A., Lee X., Torrence P.F., Silverman R.H. Intrinsic molecular activities of the interferon-induced 2–5A-dependent RNase. J. Biol. Chem. 1994; 269:14153–14158. 7514601

[10] Hovanessian A.G., Justesen J. The human 2′-5′-oligoadenylate synthetase family: Unique interferon-inducible enzymes catalyzing 2′-5′ instead of 3′-5′ phosphodiester bond formation. Biochimie. 2007; 89:779–788. 17408844 10.1016/j.biochi.2007.02.003

[11] Han Y., Whitney G., Donovan J., Korennykh A. Innate immune messenger 2–5A tethers human RNase L into active high-order complexes. Cell Rep. 2012; 2:902–913. 23084743 10.1016/j.celrep.2012.09.004

[12] Huang H., Zeqiraj E., Dong B., Jha B.K., Duffy N.M., Orlicky S., Thevakumaran N., Talukdar M., Pillon M.C., Ceccarelli D.F. et al. Dimeric structure of pseudokinase RNase L bound to 2–5A reveals a basis for interferon-induced antiviral activity. Mol. Cell. 2014; 53:221–234. 24462203 10.1016/j.molcel.2013.12.025 PMC3974923

[13] Malathi K., Dong B., Gale M. Jr, Silverman R.H. Small self-RNA generated by RNase L amplifies antiviral innate immunity. Nature. 2007; 448:816–819. 17653195 10.1038/nature06042 PMC3638316

[14] Banerjee S., Li G., Li Y., Gaughan C., Baskar D., Parker Y., Lindner D.J., Weiss S.R., Silverman R.H. RNase L is a negative regulator of cell migration. Oncotarget. 2015; 6:44360–44372. 26517238 10.18632/oncotarget.6246 PMC4792562

[15] Rath S., Donovan J., Whitney G., Chitrakar A., Wang W., Korennykh A. Human RNase L tunes gene expression by selectively destabilizing the microRNA-regulated transcriptome. Proc. Natl Acad. Sci. U.S.A. 2015; 112:15916–15921. 26668391 10.1073/pnas.1513034112 PMC4702973

[16] Siddiqui M.A., Mukherjee S., Manivannan P., Malathi K. RNase L cleavage products promote switch from autophagy to apoptosis by caspase-mediated cleavage of Beclin-1. Int. J. Mol. Sci. 2015; 16:17611–17636. 26263979 10.3390/ijms160817611 PMC4581211

[17] Donovan J., Rath S., Kolet-Mandrikov D., Korennykh A. Rapid RNase L-driven arrest of protein synthesis in the dsRNA response without degradation of translation machinery. RNA. 2017; 23:1660–1671. 28808124 10.1261/rna.062000.117 PMC5648034

[18] Chitrakar A., Rath S., Donovan J., Demarest K., Li Y., Sridhar R.R., Weiss S.R., Kotenko S.V., Wingreen N.S., Korennykh A. Real-time 2–5A kinetics suggest that interferons beta and lambda evade global arrest of translation by RNase L. Proc. Natl. Acad. Sci. U.S.A. 2019; 116:2103–2111. 30655338 10.1073/pnas.1818363116 PMC6369740

[19] Wreschner D.H., McCauley J.W., Skehel J.J., Kerr I.M. Interferon action–sequence specificity of the ppp(A2′p)nA-dependent ribonuclease. Nature. 1981; 289:414–417. 6162102 10.1038/289414a0

[20] Malathi K., Saito T., Crochet N., Barton D.J., Gale M. Jr, Silverman R.H. RNase L releases a small RNA from HCV RNA that refolds into a potent PAMP. RNA. 2010; 16:2108–2119. 20833746 10.1261/rna.2244210 PMC2957051

[21] Cayley P.J., Davies J.A., McCullagh K.G., Kerr I.M. Activation of the ppp(A2′p)nA system in interferon-treated, herpes simplex virus-infected cells and evidence for novel inhibitors of the ppp(A2′p)nA-dependent RNase. Eur. J. Biochem. 1984; 143:165–174. 6088228 10.1111/j.1432-1033.1984.tb08355.x

[22] Drappier M., Jha B.K., Stone S., Elliott R., Zhang R., Vertommen D., Weiss S.R., Silverman R.H., Michiels T. A novel mechanism of RNase L inhibition: Theiler's virus L* protein prevents 2–5A from binding to RNase L. PLoS Path. 2018; 14:e1006989. 10.1371/journal.ppat.1006989 PMC5927464 29652922

[23] Han J.Q., Barton D.J. Activation and evasion of the antiviral 2′-5′ oligoadenylate synthetase/ribonuclease L pathway by hepatitis C virus mRNA. RNA. 2002; 8:512–525. 11991644 10.1017/s1355838202020617 PMC1370272

[24] Keel A.Y., Jha B.K., Kieft J.S. Structural architecture of an RNA that competitively inhibits RNase L. RNA. 2012; 18:88–99. 22114318 10.1261/rna.030007.111 PMC3261747

[25] Min J.Y., Krug R.M. The primary function of RNA binding by the influenza A virus NS1 protein in infected cells: Inhibiting the 2′-5′ oligo (A) synthetase/RNase L pathway. Proc. Natl Acad. Sci. U.S.A. 2006; 103:7100–7105. 16627618 10.1073/pnas.0602184103 PMC1459024

[26] Silverman R.H. Viral encounters with 2′,5′-oligoadenylate synthetase and RNase L during the interferon antiviral response. J. Virol. 2007; 81:12720–12729. 17804500 10.1128/JVI.01471-07 PMC2169107

[27] Silverman R.H., Weiss S.R. Viral phosphodiesterases that antagonize double-stranded RNA signaling to RNase L by degrading 2–5A. J. Interferon Cytokine Res. 2014; 34:455–463. 24905202 10.1089/jir.2014.0007 PMC4046343

[28] Taguchi T., Nagano-Fujii M., Akutsu M., Kadoya H., Ohgimoto S., Ishido S., Hotta H. Hepatitis C virus NS5A protein interacts with 2′,5′-oligoadenylate synthetase and inhibits antiviral activity of IFN in an IFN sensitivity-determining region-independent manner. J. Gen. Virol. 2004; 85:959–969. 15039538 10.1099/vir.0.19513-0

[29] Thornbrough J.M., Jha B.K., Yount B., Goldstein S.A., Li Y., Elliott R., Sims A.C., Baric R.S., Silverman R.H., Weiss S.R. Middle East Respiratory Syndrome Coronavirus NS4b protein inhibits host RNase L activation. mBio. 2016; 7:e00258. 27025250 10.1128/mBio.00258-16 PMC4817253

[30] Townsend H.L., Jha B.K., Han J.Q., Maluf N.K., Silverman R.H., Barton D.J. A viral RNA competitively inhibits the antiviral endoribonuclease domain of RNase L. RNA. 2008; 14:1026–1036. 18426919 10.1261/rna.958908 PMC2390801

[31] Townsend H.L., Jha B.K., Silverman R.H., Barton D.J. A putative loop E motif and an H-H kissing loop interaction are conserved and functional features in a group C enterovirus RNA that inhibits ribonuclease L. RNA Biol. 2008; 5:263–272. 19088502 10.4161/rna.7165 PMC2953469

[32] Zhang R., Jha B.K., Ogden K.M., Dong B., Zhao L., Elliott R., Patton J.T., Silverman R.H., Weiss S.R. Homologous 2′,5′-phosphodiesterases from disparate RNA viruses antagonize antiviral innate immunity. Proc. Natl Acad. Sci. U.S.A. 2013; 110:13114–13119. 23878220 10.1073/pnas.1306917110 PMC3740845

[33] Zhao L., Jha B.K., Wu A., Elliott R., Ziebuhr J., Gorbalenya A.E., Silverman R.H., Weiss S.R. Antagonism of the interferon-induced OAS-RNase L pathway by murine coronavirus ns2 protein is required for virus replication and liver pathology. Cell Host Microb. 2012; 11:607–616. 10.1016/j.chom.2012.04.011 PMC3377938 22704621

[34] Yakub I., Lillibridge K.M., Moran A., Gonzalez O.Y., Belmont J., Gibbs R.A., Tweardy D.J. Single nucleotide polymorphisms in genes for 2′-5′-oligoadenylate synthetase and RNase L inpatients hospitalized with West Nile virus infection. J. Infect. Dis. 2005; 192:1741–1748. 16235172 10.1086/497340

[35] Lim J.K., Lisco A., McDermott D.H., Huynh L., Ward J.M., Johnson B., Johnson H., Pape J., Foster G.A., Krysztof D. et al. Genetic variation in OAS1 is a risk factor for initial infection with West Nile virus in man. PLoS Path. 2009; 5:e1000321. 10.1371/journal.ppat.1000321 PMC2642680 19247438

[36] Bigham A.W., Buckingham K.J., Husain S., Emond M.J., Bofferding K.M., Gildersleeve H., Rutherford A., Astakhova N.M., Perelygin A.A., Busch M.P. et al. Host genetic risk factors for West Nile virus infection and disease progression. PLoS One. 2011; 6:e24745. 21935451 10.1371/journal.pone.0024745 PMC3174177

[37] Field L.L., Bonnevie-Nielsen V., Pociot F., Lu S., Nielsen T.B., Beck-Nielsen H. OAS1 splice site polymorphism controlling antiviral enzyme activity influences susceptibility to type 1 diabetes. Diabetes. 2005; 54:1588–1591. 15855350 10.2337/diabetes.54.5.1588

[38] Fedetz M., Matesanz F., Caro-Maldonado A., Fernandez O., Tamayo J.A., Guerrero M., Delgado C., Lopez-Guerrero J.A., Alcina A. OAS1 gene haplotype confers susceptibility to multiple sclerosis. Tissue Antigens. 2006; 68:446–449. 17092260 10.1111/j.1399-0039.2006.00694.x

[39] O’Brien M., Lonergan R., Costelloe L., O’Rourke K., Fletcher J.M., Kinsella K., Sweeney C., Antonelli G., Mills K.H., O’Farrelly C. et al. OAS1: a multiple sclerosis susceptibility gene that influences disease severity. Neurology. 2010; 75:411–418. 20679634 10.1212/WNL.0b013e3181ebdd2b

[40] Mandal S., Abebe F., Chaudhary J. 2′-5′ oligoadenylate synthetase 1 polymorphism is associated with prostate cancer. Cancer. 2011; 117:5509–5518. 21638280 10.1002/cncr.26219 PMC3167978

[41] Li H., Reksten T.R., Ice J.A., Kelly J.A., Adrianto I., Rasmussen A., Wang S., He B., Grundahl K.M., Glenn S.B. et al. Identification of a Sjogren's syndrome susceptibility locus at OAS1 that influences isoform switching, protein expression, and responsiveness to type I interferons. PLoS Genet. 2017; 13:e1006820. 28640813 10.1371/journal.pgen.1006820 PMC5501660

[42] Liu X., Xing H., Gao W., Yu D., Zhao Y., Shi X., Zhang K., Li P., Yu J., Xu W. et al. A functional variant in the OAS1 gene is associated with Sjogren's syndrome complicated with HBV infection. Sci. Rep. 2017; 7:17571. 29242559 10.1038/s41598-017-17931-9 PMC5730593

[43] Wu S., Wang Y., Chen G., Zhang M., Wang M., He J.Q. 2′-5′-Oligoadenylate synthetase 1 polymorphisms are associated with tuberculosis: a case-control study. BMC Pulm. Med. 2018; 18:180. 30497421 10.1186/s12890-018-0746-x PMC6267069

[44] Kondratova A.A., Cheon H., Dong B., Holvey-Bates E.G., Hasipek M., Taran I., Gaughan C., Jha B.K., Silverman R.H., Stark G.R. Suppressing PARylation by 2′,5′-oligoadenylate synthetase 1 inhibits DNA damage-induced cell death. EMBO J. 2020; 39:e101573. 32323871 10.15252/embj.2019101573 PMC7265237

[45] Banerjee S., Gusho E., Gaughan C., Dong B., Gu X., Holvey-Bates E., Talukdar M., Li Y., Weiss S.R., Sicheri F. et al. OAS-RNase L innate immune pathway mediates the cytotoxicity of a DNA-demethylating drug. Proc. Natl Acad. Sci. U.S.A. 2019; 116:5071–5076. 30814222 10.1073/pnas.1815071116 PMC6421468

[46] Hartmann R., Justesen J., Sarkar S.N., Sen G.C., Yee V.C. Crystal structure of the 2′-specific and double-stranded RNA-activated interferon-induced antiviral protein 2′-5′-oligoadenylate synthetase. Mol. Cell. 2003; 12:1173–1185. 14636576 10.1016/s1097-2765(03)00433-7

[47] Donovan J., Dufner M., Korennykh A. Structural basis for cytosolic double-stranded RNA surveillance by human oligoadenylate synthetase 1. Proc. Natl Acad. Sci. U.S.A. 2013; 110:1652–1657. 23319625 10.1073/pnas.1218528110 PMC3562804

[48] Lohofener J., Steinke N., Kay-Fedorov P., Baruch P., Nikulin A., Tishchenko S., Manstein D.J., Fedorov R. The activation mechanism of 2′-5′-oligoadenylate synthetase gives new insights into OAS/cGAS triggers of innate immunity. Structure. 2015; 23:851–862. 25892109 10.1016/j.str.2015.03.012

[49] Hartmann R., Norby P.L., Martensen P.M., Jorgensen P., James M.C., Jacobsen C., Moestrup S.K., Clemens M.J., Justesen J. Activation of 2′-5′ oligoadenylate synthetase by single-stranded and double-stranded RNA aptamers. J. Biol. Chem. 1998; 273:3236–3246. 9452437 10.1074/jbc.273.6.3236

[50] Kodym R., Kodym E., Story M.D. 2′-5′-Oligoadenylate synthetase is activated by a specific RNA sequence motif. Biochem. Biophys. Res. Commun. 2009; 388:317–322. 19665006 10.1016/j.bbrc.2009.07.167

[51] Vachon V.K., Calderon B.M., Conn G.L. A novel RNA molecular signature for activation of 2′-5′ oligoadenylate synthetase-1. Nucleic Acids Res. 2015; 43:544–552. 25477390 10.1093/nar/gku1289 PMC4288181

[52] Calderon B.M., Conn G.L. A human cellular noncoding RNA activates the antiviral protein 2′-5′-oligoadenylate synthetase 1. J. Biol. Chem. 2018; 293:16115–16124. 30126839 10.1074/jbc.RA118.004747 PMC6187638

[53] Nielsen S., Yuzenkova Y., Zenkin N. Mechanism of eukaryotic RNA polymerase III transcription termination. Science. 2013; 340:1577–1580. 23812715 10.1126/science.1237934 PMC3760304

[54] Koul A., Deo S., Booy E.P., Orriss G.L., Genung M., McKenna S.A. Impact of double-stranded RNA characteristics on the activation of human 2′-5′-oligoadenylate synthetase 2 (OAS2). Biochem. Cell. Biol. 2020; 98:70–82. 30965010 10.1139/bcb-2019-0060

[55] Justesen J., Kjeldgaard N.O. Spectrophotometric pyrophosphate assay of 2′,5′-oligoadenylate synthetase. Anal. Biochem. 1992; 207:90–93. 1336938 10.1016/0003-2697(92)90506-3

[56] Donovan J., Whitney G., Rath S., Korennykh A. Structural mechanism of sensing long dsRNA via a noncatalytic domain in human oligoadenylate synthetase 3. Proc. Natl Acad. Sci. U.S.A. 2015; 112:3949–3954. 25775560 10.1073/pnas.1419409112 PMC4386348

[57] Wang Y., Holleufer A., Gad H.H., Hartmann R. Length dependent activation of OAS proteins by dsRNA. Cytokine. 2019; 126:154867. 31629990 10.1016/j.cyto.2019.154867

[58] Marie I., Blanco J., Rebouillat D., Hovanessian A.G. 69-kDa and 100-kDa isoforms of interferon-induced (2′-5′)oligoadenylate synthetase exhibit differential catalytic parameters. Eur. J. Biochem. 1997; 248:558–566. 9346316 10.1111/j.1432-1033.1997.t01-1-00558.x

[59] Charley P.A., Wilusz J. Sponging of cellular proteins by viral RNAs. Curr. Opin. Virol. 2014; 9:14–18. 25233339 10.1016/j.coviro.2014.09.001 PMC4268051

[60] Li S., Olson W.K., Lu X.J. Web 3DNA 2.0 for the analysis, visualization, and modeling of 3D nucleic acid structures. Nucleic Acids Res. 2019; 47:W26–w34. 31114927 10.1093/nar/gkz394 PMC6602438

